# How Arrestins and GRKs Regulate the Function of Long Chain Fatty Acid Receptors

**DOI:** 10.3390/ijms232012237

**Published:** 2022-10-13

**Authors:** Abdulrahman G. Alharbi, Andrew B. Tobin, Graeme Milligan

**Affiliations:** 1 Centre for Translational Pharmacology, School of Biomolecular Sciences, College of Medical, Veterinary and Life Sciences, University of Glasgow, Glasgow G12 8QQ, UK; 2 Department of Pharmacology and Toxicology, College of Pharmacy, Taibah University, Madinah 42353, Saudi Arabia

**Keywords:** arrestins, GRKs, FFA1, FFA4

## Abstract

FFA1 and FFA4, two G protein-coupled receptors that are activated by long chain fatty acids, play crucial roles in mediating many biological functions in the body. As a result, these fatty acid receptors have gained considerable attention due to their potential to be targeted for the treatment of type-2 diabetes. However, the relative contribution of canonical G protein-mediated signalling versus the effects of agonist-induced phosphorylation and interactions with β-arrestins have yet to be fully defined. Recently, several reports have highlighted the ability of β-arrestins and GRKs to interact with and modulate different functions of both FFA1 and FFA4, suggesting that it is indeed important to consider these interactions when studying the roles of FFA1 and FFA4 in both normal physiology and in different disease settings. Here, we discuss what is currently known and show the importance of understanding fully how β-arrestins and GRKs regulate the function of long chain fatty acid receptors.

## 1. Introduction: Free Fatty Acids (FFAs)

**Figure 1:**
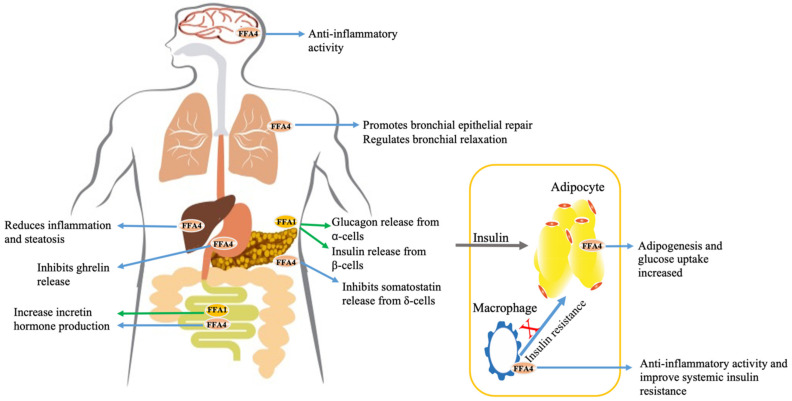
**Activities that may be regulated by FFA1 and FFA4 receptor ligands**. Release of glucagon from pancreatic α-cells and insulin from β-cells is induced by FFA1 that couples to both G_q_/_11_ and β-arrestin-dependent pathways. FFA1 in enteroendocrine cells regulates the secretion of incretin hormones including Glucagon-Like Peptide-1 and cholecystokinin. FFA4 is also present in enteroendocrine cells, as well as in lung, brain, white adipose tissue, and the liver. Within adipose tissue, a rise in adipogenesis and glucose uptake is associated with activation of FFA4. Anti-inflammatory benefits are mostly attributed to the ability of FFA4 to recruit β-arrestin-2 in an agonist-dependent manner and its subsequent consequences. Green arrows illustrate the function of FFA1 receptors and blue arrows illustrate the function of FFA4 receptors in the specified tissue site.

There has recently been a substantial expansion of knowledge concerning the ability of chemicals found in, or generated from, dietary sources to manage and govern cellular activity and maintain homeostasis [1,2,3]. Amongst such molecules non-esterified or “free” fatty acids (FFAs) have long been recognised as having various impacts on numerous biological systems, including cardiovascular health, metabolism, and inflammation [4]. Although it was previously thought that fatty acids exerted such actions exclusively via intracellular targets, it has subsequently been shown that they also stimulate a number of cell surface G protein-coupled receptors (GPCRs). It has long been a staple of drug development efforts to target GPCRs [5] and therefore GPCRs activated by FFAs have become of major interest as potential novel therapeutic targets [6,7,8]. Indeed, programmes targeting the receptors for FFAs have investigated the actions of each of orthosteric and allosteric modulators to try to optimise therapeutic activity [9,10]. Major efforts have examined these receptors in relation to metabolism and inflammation, in particular the role of FFA1 and FFA4 in the management of type-2 diabetes [7,8,11,12]. Other studies have also indicated that they play roles in areas ranging from lung function to the central nervous system [13,14,15]. Currently four GPCRs, FFA1-FFA4, are considered as authentic free fatty acid receptors, and whilst GPR84 remains officially an orphan receptor [16], it is nevertheless widely known that medium chain length (C9–C12) fatty acids are able to activate this receptor, indicating it to be a further FFA responsive GPCR. Short chain fatty acids.

(C2–C6) are selective activators of FFA2 and FFA3 whilst longer chain fatty acids are effective agonists for FFA1 and FFA4 [4,17,18]. Both FFA1 and FFA4 are extensively expressed in a variety of tissues and regions of the body and participate in vital physiological functions that are closely connected to metabolism and immunity [12] (Figure 1). Many pharmaceutical companies have initiated studies to design and test ligands that stimulate FFA1 and FFA4 due to the role of these receptors in the control of blood glucose with the ambition to treat the increasing number of T2D patients. The focus of this review will hence be on FFA1 and FFA4, the receptors for longer chain fatty acids, and on how their function is regulated by GPCR kinases (GRKs) and by arrestins.

### Free Fatty Acid Receptor 1

**Figure 2:**
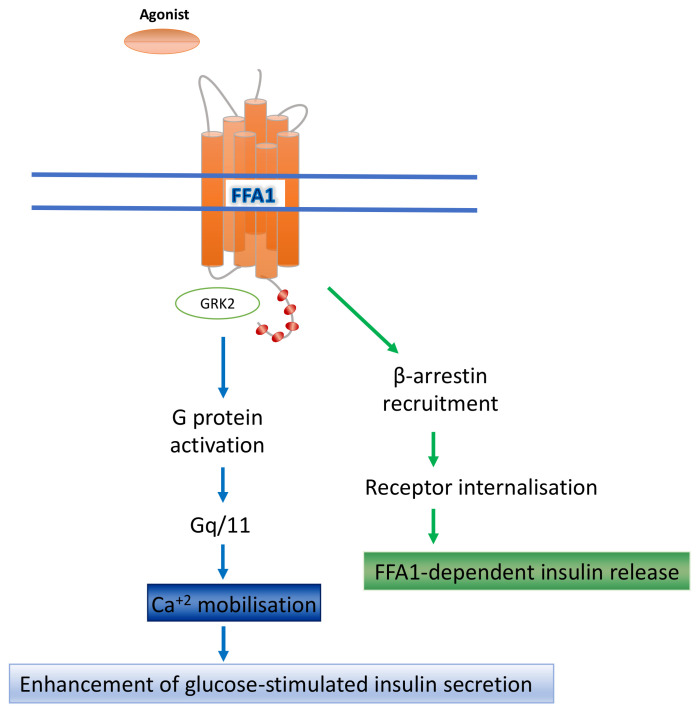
**Major FFA1 signalling pathways**. Through G protein (Gq/11)- and β-arrestin-dependent pathways stimulation of FFA1 increases insulin production and improves insulin sensitivity. Interactions with GRKs (here illustrated as GRK2) may alter the balance between G protein and β-arrestin-mediated signalling. Numerous attempts to develop synthetic ligands at FFA1 have employed receptor-β-arrestin interaction studies within the workflow. Various studies suggest interactions regulated by β-arrestin interaction control insulin release from pancreatic β-cells, although additional studies suggest that there is an interplay of both G protein and β-arrestin routes in response to activation by FFA1 ligands [34].

Still frequently referred to as GPR40, the Free Fatty Acid Receptor 1 (FFA1), which was first described merely as an uncharacterised GPCR sequence found in humans at chromosome location 19q13.1, is expressed in high levels by pancreatic β-cells. Three distinct investigations reported in 2003 showed this receptor could be stimulated by medium and long chain fatty acids [19,20,21]. A variety of saturated and unsaturated fatty acids activate the receptor and results in an increase in intracellular Ca^2+^ levels. Several studies employed the selective G_q_/_11_ inhibitor YM-254890 [22] to establish that such signals from FFA1 stimulation are transmitted mostly through G_q_/_11_-family G proteins [23]. Recent years have also seen the use of FR900359 [24], a second, related depsipeptide G_q_/_11_ inhibitor, to corroborate these findings. Many FFA1-active ligands have been developed and reported (Table 1), and a range of studies have been conducted in relation to the potential treatment of many physiological and metabolic illnesses, including cardiovascular diseases, type-2 diabetes, obesity, atherosclerosis, ulcerative colitis, Crohn’s disease, and irritable bowel syndrome [25,26]. Among these disorders, the activity of FFA1 in promoting the release of insulin from pancreatic β-cells in a glucose concentration-dependent manner has attracted the greatest attention [27,28]. At least in part this effect on insulin release reflects receptor-induced enhancement of intracellular calcium signalling [29,30,31] whereby intracellular Ca^2+^ levels rise as a result of G_q_/G_11_-mediated production of the secondary messenger inositol 1,4,5 trisphosphate (IP3). However, glucose-stimulated insulin secretion (GSIS) from pancreatic β-cells not only requires the presence of glucose but also requires other signalling stimuli that are activated by external calcium [20,32,33]. Examples of these stimuli include peptide hormones, neurotransmitters, and other compounds [8]. Although G protein-dependent pathways are recognised to be the primary mechanism by which FFA1 mediates signal transduction [28], there is nevertheless another signalling route that FFA1 may activate (Figure 2), where function is reliant on arrestin adapter proteins [34]. Indeed insulin secretion is connected to an FFA1-arrestin signalling axis [35].

Both orthosteric and allosteric FFA1 agonists have been identified and developed and these may differentially regulate FFA1 activity, resulting in a signalling bias [35]. Fatty acids, such as palmitic acid (C16:0) and oleic acid (C18:1), generate G_q_/_11_-mediated signalling via FFA1; however, they also recruit β-arrestin 2 and promote internalisation of FFA1 from the cell surface [34]. β-Arrestin recruitment to FFA1 originates via both G_q_/_11_-dependent and -independent processes with the balance between these functions induced by distinct activators that may define their effectiveness [34]. For example, both palmitic acid and oleic acid have insulinotropic effects, mostly due to G_q_/_11_ mediated pathways. However, the clinically trialled synthetic allosteric agonist, TAK-875 demonstrated a distinct relative efficacy for activating the G_q_/_11_ and β-arrestin routes. Here it is only a partial agonist for G_q_/_11_ activation whilst the fatty acids palmitate and oleate may be less efficient than TAK-875 for the recruitment of β-arrestins [34]. To improve insulin production in people with type-2 diabetes, targeting the biased stimulation of FFA1 has shown promising results [15]. However phase III clinical trials with TAK-875 were terminated because of indications of liver toxicity in patients [36]. As a result, although treatments based on FFA1 agonism may still provide a promising option in the search for new anti-diabetic medications, and a number of additional ligands have already been assessed in rodent models, there has nevertheless been limited progression towards human studies beyond initial phase I safety studies (e.g., [37]). Importantly, recent research has revealed the remarkable complexity of FFA1 ligand actions providing optimism that with a better knowledge of the underlying processes the next phase will be more effective [29,38]. Additional studies include phase I data on the oral FFA1 inhibitor P11187 [12] but, whilst completing phase II clinical trials, the development of JTT-851, a further orally active FFA1 agonist, was also terminated [39]. Further molecules that have shown interesting characteristics in rodent models include the high potency FFA1 agonist TUG-770, which was able to increase glucose tolerance in high fat diet fed mice [40] and DS-1558 which improved insulin production and glucose homeostasis in ‘Zucker’ diabetic fatty rats [40]. Other compounds such as AM-1638 and AM-5262 can additionally engage further signalling cascades including the G_s_ G protein [41,42,43]. These compounds enhance incretin production from enteroendocrine cells and directly stimulate insulin production whilst AP1 and AP3, both of which are reported as full agonists of FFA1, promote insulin and incretin production in diabetic ‘Goto-Kakizaki’ rats, resulting in a reduction in both body mass and blood sugar levels [43,44]. Recently, Mach et al. have reported CPL207280 which, in their in vitro studies, was more effective than TAK-875 [37]. These examples highlight the varying signalling characteristics of different FFA1 activators and the pathways with which the receptor can engage. Whether the optimal characteristics of ligands targeting FFA1 that may have beneficial effects on diabetic patients can be defined and incorporated into ligands that lack the limitations of TAK-875 in terms of bile acid transporter function remain to be ascertained.

**Table 1:** Summary of synthetic ligand of long chain free fatty acids pharmacology.

Ligand	Mode of Action	Usage	Status	References
**FFA1 agonists**				
TAK-875	Stimulate intracellular Ca^2+^ mobilisation which result in increased blood insulin levels and reduces fasting hyperglycaemia	Type-2 diabetes	Terminated due to liver toxicity	[45,46,47]
TUG-770	Stimulate intracellular Ca^2+^ mobilisation which result in increased blood insulin levels	Type-2 diabetes	Preclinical studies on animals	[40]
TUG-424	Stimulate intracellular Ca^2+^ mobilisation leading to an increase in insulin secretion from pancreatic islets	Type-2 diabetes	Preclinical studies on animals	[48,49]
TUG-499	Stimulate intracellular Ca^2+^ mobilisation leading to an increase in insulin secretion from pancreatic islets	Type-2 diabetes	Preclinical studies on animals	[48,50]
TUG-488	Stimulate intracellular Ca^2+^ mobilisation leading to an increase in insulin secretion from pancreatic islets	Type-2 diabetes	Preclinical studies on animals	[50]
TUG-469	Stimulate intracellular Ca^2+^ mobilisation which significantly improved glucose tolerance	Type-2 diabetes	Preclinical studies on animals	[51]
DS-1558	Stimulate intracellular Ca^2+^ mobilisation which significantly improved glucose tolerance insulin secretion from pancreatic islets	Type-2 diabetes	Preclinical studies on animals	[41]
GW9508	Stimulate intracellular Ca^2+^ mobilisation leading to stimulation of insulin release from pancreatic islets	Type-2 diabetes	Preclinical studies on animals	[52]
LY2881835	Stimulate intracellular Ca^2+^ mobilisation Immediate and prolonged increase of GLP-1 secretion	Type-2 diabetes	Stopped after phase I trial	[53,54,55]
AM-1638	Stimulate intracellular Ca^2+^ mobilisation leading to stimulation of insulin release from pancreatic islets and an increase in GLP-1 secretion from the gut	Stimulate insulin secretion and promote incretin release from enteroendocrine cells	Preclinical studies on animals	[49,56,57,58]
AM-5262	Stimulate intracellular Ca^2+^ mobilisation leading to stimulation of insulin release from pancreatic islets and an increase in GLP-1 secretion from the gut	Stimulate insulin secretion and promote incretin release from enteroendocrine cells	Preclinical studies on animals	[49,56,57,58]
**FFA1 antagonists**				
GW1100	Inhibiting cell motility	Osteosarcoma, pituitary cultures, colon cancer cells and enteroendocrine L-cells	Preclinical studies on animals	[59,60,61,62]
ANT203	Inhibition of FFA1 receptor function during the long-term exposure to palmitate resulting in low fatty acid oxidation and positive effects on pancreatic β-cell function	Protects against β-cell dysfunction and apoptosis induced by chronic FFA exposure	Preclinical studies on animals	[63]
DC260126	Improve insulin sensitivity and protect pancreatic β-cells from apoptosis in diabetic mice	Treatment of type-2 diabetes	Preclinical studies on animals	[64,65]
**FFA4 agonists**				
GW9508	Blocking extracellular signal-regulated kinase activity	Prevent fasting-induced plasma ghrelin elevation	Preclinical studies on animals	[66]
NCG21	Activates extracellular signal-regulated kinase Stimulate intracellular Ca^2+^ level	Increase plasma GLP-1 levels	Preclinical studies on animals	[67]
TUG-891	Stimulate intracellular Ca^2+^ mobilisation β-arrestin recruitment Activates extracellular signal-regulated kinase	Ameliorate inflammation in visceral white adipose tissue and insulin resistance Enhance fat oxidation and reduce fat mass in mice Osteoporosis	Preclinical studies on animals	[68,69,70]
TUG-1197	Stimulate intracellular Ca^2+^ level β-arrestin recruitment	Type-2 diabetes Inflammatory disorders	Preclinical studies on animals	[71]
GSK137647A	Suppress the adipogenic differentiation of bone mesenchymal stem cells Inhibiting palmitic acid-induced elevation of proinflammatory chemokines and activation of NF-κB, JNK1/2, and p38MAPK signalling pathways	Osteogenic and adipogenic differentiation of bone mesenchymal stem cells Type-2 diabetes	Preclinical studies on animals	[72,73]
Merck Compound A	β-arrestin recruitment Stimulate intracellular Ca^2+^ mobilisation	Anti-inflammatory effects in macrophages Type-2 diabetes	Preclinical studies on animals	[14,74]
Metabolex 36	Activation of Gαi/o	Study FFA4 function in pancreatic δ cells	Preclinical studies on animals	[75]
Metabolex B	Activation of Gαi/o	Role of FFA4 in ghrelin secretion and somatostatin release from mouse pancreas	Preclinical studies on animals	[76,77]
KDT501	Help in control ling impaired glucose tolerance and insulin sensitivity in patient with insulin resistance	Type-2 diabetes	Preclinical studies on animals	[78]
**FFA4 antagonists**				
AH7614	Inhibit the intracellular release of Ca^2+^	Type-2 diabetes Interstitial cystitis syndrome Non-alcoholic fatty liver disease	Preclinical studies on animals	[79,80,81]

## 2. Free Fatty Acid Receptor 4

Additionally, known as GPR120, FFA4 was de-orphanised as an FFA receptor almost 20 years ago [4,18,82,83]. It responds to a similar group of long chain FFAs as FFA1, and is also a member of the rhodopsin subgroup of GPCRs [83]. FFA4 has been identified in different tissues and may serve different physiological roles [84,85] (Figure 1). FFA4 is expressed abundantly in the intestinal tract [18,86] and Lu et al. (2021) and Hirasawa and co-workers (2005) demonstrated that the stimulation of FFA4 promotes release of glucagon-like peptide-1 (GLP-1) from gut cells [18,87]. However, the importance of this receptor to this end-point remains contentious [8,14,85,87,88]. In the duodenum, FFA4 shows high co-localisation with the satiety hormone ghrelin [89]. FFA4 is also expressed in other specific cell types, such as K cells and brush cells. In K cells it promotes the release of insulin [87] whilst in brush cells, FFA4 was found at the limited ridge of the mouse stomach, making FFA4 a reasonable candidate to sense long chain fatty acids in the lumen [90]. Expression of FFA4 in other tissues, including lung, heart and skeletal muscle, is known but the physiological roles of FFA4 in these tissues need further study before they can be fully understood. However, in recent years its roles in the lung [13] and in δ cells of the pancreas [14] have been investigated. In addition, FFA4 knockout mice develop obesity when fed a high-fat diet and glucose intolerance and fatty livers were noted in such animals lacking FFA4 [91]. Such observations have increased the motivation to study FFA4, often in the context of diabetes and other metabolic syndromes [71]. In the treatment of type-2 diabetes, FFA4 ligands have attracted considerable interest because of their ability to enhance glucose-dependent insulin production from pancreatic β-cells as well as their anti-inflammatory actions in adipocytes and the gut [85] (Table 1). Stimulation of this receptor by oleic acid enhances lipid droplet formation in adipose tissues via the G_q_, PI3K-Akt, and PLC signalling pathways, while in enteroendocrine cells it regulates hormone production by promoting GLP-1 release and inhibiting ghrelin secretion [18,92,93]. Although not restricted to polyunsaturated fatty acids (PUFAs), there has been interest in whether such ligands, especially those of the ω-3 family, may regulate a distinct subset of the capabilities of the FFA4 receptor [73,93,94,95]. The development of synthetic FFA4 selective agonists has also been central to gaining a deeper knowledge of the biological actions of this receptor. Many of the initially described FFA4 active ligands were closely related to fatty acids, and ligands such as GW9508 and NCG21, show only a modest selectivity for FFA4 over FFA1 [65,96,97]. The first potent and selective FFA4 agonist was TUG-891 [98] and this is still by far the most widely used ligand in the literature. Moreover, the phenylpropionic acid core of this molecule has been the inspiration for a wide range of subsequently reported FFA4 active and selective agonists [4,8]. Despite being a potent and selective FFA4-selective ligand in humans, TUG-891 displays a lower selectivity between the mouse orthologues of FFA4 and FFA1 [99], and this needs to be considered when using mouse models of disease. Other FFA4 ligands that have been used relatively widely in the literature include GSK137647A [72], Merck Compound A [14], Metabolex 36 and Metabolex compound B [77], and TUG-1197 [71].

**Figure 3:**
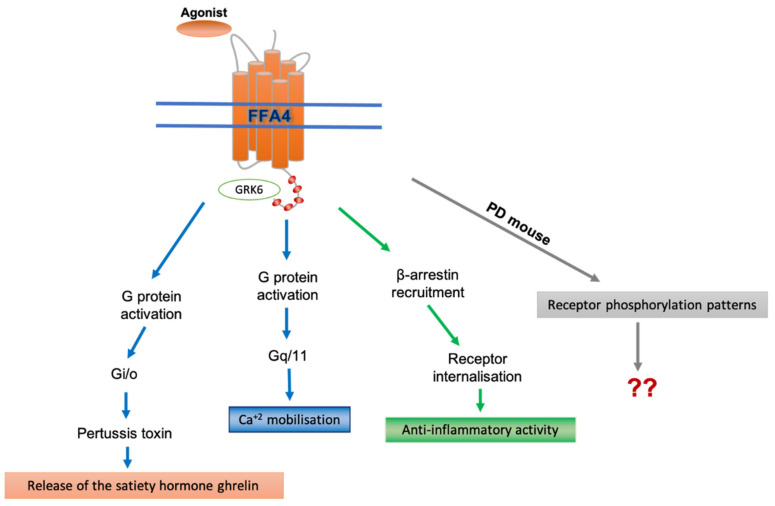
**FFA4 engages with a variety of pathways to control signaling and physiological functions**. Ligand-induced interactions of FFA4 with G_q_/G_11_ G proteins leads to increased intracellular [Ca^2+^] levels. This pathway is fundamental to many of the effects that FFA4 has in physiological settings. Numerous efforts to develop synthetic ligands of FFA4 have employed receptor-β-arrestin interaction assays. A key physiological function of FFA4 engagement with a β-arrestin is the production of anti-inflammatory mediators by macrophages. A number of studies have defined the key sites of agonist-mediated, GRK-dependent (here shown as GRK6) phosphorylation in both human and mouse FFA4 (see text). A transgenic knock-in mouse line expressing a phosphorylation-deficient (PD mouse) form of FFA4 is available [82,96] and this will help to assess the specific roles and functions of phosphorylation of FFA4, including in mouse models of disease. Studies have also shown an important role for FFA4 interactions with pertussis-toxin-sensitive G proteins to control the release of the satiety hormone ghrelin [76].

Ligands acting on FFA4 promote receptor interactions with G_q_/_11_ and hence activate phospholipase C, and boost Ca^2+^ levels (Figure 3), which ultimately results in the release of L cell-expressed peptide hormones [100]. As with FFA1, that selective G_q_/G_11_ blockers, such as FR900359 and YM-254890 are able to ablate such signals, demonstrates the significance of this G protein subfamily in the fundamental components of FFA4 actions [99]. Moreover, this is supported by the fact that FFA4 lacked the ability to increase inositol phosphates or intracellular Ca^2+^ concentrations when expressed in HEK293 cells in which the expression of both G_q_ and G_11_ that had been eliminated by genome editing [101]. This signalling route is fundamental to many of the effects that FFA4 has in physiological settings [8,82,101,102]. On the other hand, treatment with pertussis toxin, which defines a function for G_i_-family G proteins, eliminates FFA4-mediated production of the hormone ghrelin [76]. It is clear that agonist-activated FFA4 interacts rapidly, and in a sustained fashion, with β-arrestins and this results in the rapid desensitisation of G protein-mediated functions and in receptor internalisation [99]. These observations pose questions about the potential challenges in targeting FFA4 via therapeutically [100] that remain to be fully understood and resolved. Although a large body of data indicates that the activation of FFA4 can have profound anti-inflammatory effects the basis of these remains uncertain. This is despite effects in macrophages clearly appearing to involve a cascade initiated by the β-arrestin-mediated scaffolding of adaptor proteins [74,94]. The importance of arrestin-mediated signalling may indeed be cell type-dependent. This has been seen by Alvarez-Curto et al. [101] who failed be a contribution of arrestins to FFA4-mediated stimulation of ERK1/2 phosphorylation by utilising HEK293 cells that had been genome-edited to lack expression of the two β-arrestins. This was despite parental HEK293 cells often being used to implicate ERK1/2 phosphorylation as an arrestin-mediated outcome of the activation of heterologously expressed GPCRs [101]. By contrast, in such arrestin-null cells the desensitisation of G protein-mediated FFA4 signalling was abrogated, implying that the more traditional ‘arresting’ role of the β-arrestins had indeed been eliminated [101].

Although no synthetic FFA4 selective agonist has yet entered clinical trials in any disease setting [102], there have nevertheless been small scale clinical studies conducted using naturally occurring FFA4 active ligands. For example, healthy and obese patients were given pine nut oil and olive oil to consume in order to investigate the impact of these natural oils on glucose tolerance and on both FFA1, FFA4 and a further ‘metabolic’ receptor GPR119 [103,104]. Pinolenic acid, the major fatty acid component of pine nut oil is an equipotent, natural dual agonist of FFA1 and FFA4 and enhances glucose tolerance in rodents [105].

### 2.1. The GRK Family

GRKs are a class of 7 serine/threonine protein kinases that are most closely linked to the AGC kinase family. On the basis of their sequence similarities, members of the GRK family are categorised into the following three subgroups: the rhodopsin kinase or visual GRK subgroup comprising GRK1 and GRK7, the ‘β-adrenergic’ receptor kinase subgroup of GRK2 and GRK3, and the GRK4 subgroup which is comprised of GRK4, GRK5 and GRK6 [106]. Although similar in terms of targeting GPCRs, the subgroups have their own distinctive regulatory features. The expression of GRK2, 3, 5, and 6 is widespread across mammalian tissues, but expression of GRK1, 4, and 7 is limited to certain organs [107,108]. GRK4 is found in the testes, cerebellum, and kidneys [109,110] whilst GRK1 and 7 are found predominantly in the rods and cones of the retina, respectively [109]. Modularity is achieved in GRKs by the presence of a short amino-acid terminal a-helical domain (N-helix) and a variable carboxy terminal lipid-binding region [111]. The catalytic region of GRKs is located inside the regulator of the G protein trafficking homology (RH) region [112]. The regulated phosphorylation of the vast majority of GPCRs is under the stringent control of the four ubiquitously expressed GRKs [112], although the contribution of each may be receptor and cell type specific. Desensitisation, internalisation, and their functional consequences are the results of the engagement of GRKs with particular receptors. Subsequent de-phosphorylation is important to allow receptors to recycle back to the cell surface [113,114]. Variation allows diversity, with some GPCRs showing sustained intracellular trafficking, which localises the receptors to particular intracellular compartments, and this may potentially lead to a second wave of endosomal-generated signalling. Such localisation of the receptors is achieved through the GPCRs’ ability to bind to their ligands for longer periods of time [115]. Within a cellular setting, the act of GRK-binding causes active GPCRs to undergo intracellular activation at the places where they are located.

GRKs play not only a vital role as regulators but also determine the actions of β-arrestins by causing ligand-specific GPCR activation or by preferentially coupling to particular active receptor regions [107]. The field of structural biology has made significant contributions to our comprehension of the architectural changes that occur in receptors before they engage in contact with G proteins or arrestins. The ubiquitous expression of GRK2, 3, 5, and 6 has made it difficult to understand the functions that each particular GRK plays in the process of receptor activation [116]. However, the use of selective small molecule GRK inhibitors, such as compound 101 to block GRK 2/3 [117] and compound 18 to block GRK5/6 [118], siRNA/shRNA methods [116,119] or CRISPR/Cas9 approaches targeting only a specific subset of relevant GRKs [107,120], is beginning to unravel the mysteries of these topics. In addition, the use of phospho-site-specific antibodies [16,121,122] and mass spectrometry analysis of the sites of regulated phosphorylation [16,96,122,123] are also providing valuable insights. For example, Marsango et al. (2022) identified a crucial pair of threonine residues in the medium chain fatty acid receptor GPR84 that are only phosphorylated in response to receptor activation, and this occurs, at least in HEK293 cells, via GRK2/3 [16]. This regulation defines efficient interactions with arrestins and allows the separation of G protein-biased and more balanced GPR84 agonists [16]. Similarly, Divorty et al. (2022) reported phospho-site-specific antisera that act as activation state-specific biomarkers for the orphan metabolite receptor GPR35 [122]. Here, pre-treatment with the GRK2/3 blocker compound 101 significantly decreased the agonist-induced phosphorylation of human, and particularly mouse, orthologues of GPR35, as detected by these antisera. These studies indicate a critical function for GRK2 and/or GRK3 on key residues to control interactions with arrestins [122].

It is not known if a particular GPCR is activated by one GRK or by numerous GRKs in a sequential and potentially hierarchical manner. Drube et al. (2022) were able to show that various GRKs and second messenger kinases are able to induce diverse outcomes based on the targeted GPCR [107]. For example, the activity of GRKs can be either increased or decreased via the action of Protein Kinase C (PKC) and the presence of G_q_-family G proteins. They recorded a decrease in GRK5- and GRK6-mediated β-arrestin recruitment to the angiotensin AT1R when PKC activity was suppressed. Clearly, however, much further analysis will still be required.

GRK2 and GRK3 are typically found within the cytosol in the absence of GPCR stimulation, but they are nevertheless able to translocate to the cell surface upon stimulation due to their engagement with the βγ-heterodimer of active G proteins [124]. By contrast, GRK5 and GRK6 are routinely membrane-localised [106]. It is possible, particularly in more complex native cells, that some receptors are found in membrane regions that are inaccessible to GRK5 and GRK6, and this may in part help shape the effects of different GRKs.

### 2.2. Arrestin 2 and Arrestin 3

There are four members of the arrestin family, namely, arrestin-2 (also known as β-arrestin 1 and arrestin-3 (β-arrestin 2), which share 78% sequence identity [125], and are widely distributed within the body. Both β-arrestin 1 and β-arrestin 2 control a variety of signalling pathways by interacting with the majority of non-visual GPCRs [112]. The potential of the β-arrestins in regulating and mediating various functions of GPCRs is well-established but the exact roles of other arrestin-like molecules, including arrestin domain-containing proteins and α-arrestins, in receptor endocytosis and other functions are not yet fully understood [126,127]. Initially β-arrestins were thought simply to function as down-regulators of GPCR signalling by preventing and ‘arresting’ GPCR g protein interactions. However, over time, a much broader set of roles in providing scaffolds for distinct signalling pathways has been uncovered [128,129]. These include the scaffolding of proteins that have a role in signal transduction pathways, including members of mitogen-activated protein kinase cascades, E3 ubiquitin ligases and Src family tyrosine kinases [129]. In terms of specificity, β-arrestin 2, but not β-arrestin 1, was reported to scaffold the ASK1-MKK4/7-JNK3 cascade in a receptor-dependent manner [112,130]. Recent research has suggested an important interplay between arrestin-stimulated signalling and GPCR engagement as it has been indicated that arrestin functioning beyond roles in desensitisation may be lacking in the absence of G protein activity [101,131].

### 2.3. Roles of GRKs and Arrestins in the Functions of FFA1 and FFA4

As described earlier, GRKs and arrestins are key adapter and scaffold molecules that can mediate the actions of a variety of distinct GPCRs [112] and, not surprisingly, both FFA1 and FFA4 have been studied in this regard. An early study was unable to record FFA1-induced β-arrestin recruitment to FFA1 [132], potentially suggesting a limited role for this in the signal transduction of FFA1. However, Qian et al. (2014) demonstrated that both β-arrestin 2 and GRK2 play roles in the linoleate-induced, clathrin-mediated internalisation of FFA1 from the surface of transfected HEK293 cells, whereas constitutive internalisation of the receptor did not utilise this route [35]. Perhaps more directly relevant to pancreatic function, knock-down or genetic elimination of β-arrestin 2 in an insulin-secreting cell line and in mouse islets, respectively, limited the insulinotropic activity of the FFA1 agonist TAK-875 [34]. Despite these studies, there has so far only been limited analysis of ligand regulated phosphorylation of FFA1. Recently Guzmán-Silva et al. (2022) performed mutagenesis on a number of serine and threonine residues in the 3rd intracellular loop and C-terminal tail of human FFA1 and observed that, although not affecting the docosahexaenoic acid (DHA)-induced elevation of Ca^2+^ when the mutants were expressed in HEK293 cells, these mutants were substantially less well phosphorylated in response to DHA compared to the wild type receptor [133]. Moreover, a mutant with alterations in both the C-terminal tail and 3rd intracellular loop was internalised very poorly [133]. Perhaps surprisingly, the characterisation of phospho-site specific antisera for FFA1 or the role of specific GRKs have not yet been reported. This is certainly not the case for FFA4: at this receptor Burns et al. (2014) used a knockdown strategy to indicate that, at least again in HEK293 cells, GRK6 was largely responsible for DHA-mediated phosphorylation of the receptor and that residues Thr^347^, Ser^350^ and Ser^357^ in the C-terminal tail were likely sites of modification [134]. These studies were rapidly complemented by Butcher et al. [135]. Using combinations of mass spectrometry, mutagenesis, and the development of phospho-site specific antisera, they showed that each of Thr^347^, Thr^349^, Ser^350^, Ser^357^, and Ser^360^ in the C-terminal tail of human FFA4 became phosphorylated in response to the synthetic FFA4 agonist TUG-891 when the receptor was expressed in either CHO or HEK293 cells. Although the roles of specific GRKs were not examined, the use of a pSer^347^-pThr^350^ site-specific antiserum showed that TUG-891-induced phosphorylation of these residues was not mediated via PKC and hence probably reflected the actions of one or more GRKs [135]. Truncation from the C-terminus to delete sites of agonist-mediated phosphorylation progressively prevented TUG-891 induced recruitment of β-arrestin 2 and receptor internalisation. Moreover, mutation to alanine of all the identified sites of agonist-induced phosphorylation restricted, but did not fully eliminate, interactions with β-arrestin 2. To do so required further substitution of acidic residues in the C-terminal tail. Replacement by alanine of no single hydroxy-amino acid was sufficient to produce a substantial effect on TUG-891-induced β-arrestin 2 recruitment, indicating the need for phosphorylation of a number of distinct residues [135]. Subsequently Prihandoko et al. explored similar themes for the mouse orthologue of FFA4 [96]. As well as showing agonist-induced phosphorylation of this orthologue at equivalent residues as in the human orthologue, these studies went on to show that mutation of only the phospho-acceptor sites at the extreme C-terminus was sufficient to limit receptor internalisation and β-arrestin 2 recruitment [96]. In contrast mutation of the more proximal residues to alanines had a limited effect on β-arrestin 2 recruitment but a substantial effect on the downstream activation of pAkt [96]. This interesting observation raises the potential for FFA4 active agonists to act in a ‘biased’ signalling fashion if they are able to phosphorylate selectively these two separable groups of Ser/Thr residues.

This concept, first described by Tobin as the ‘bar-code’ hypothesis [136], suggested that ligands, either due to their inherent nature or to variations in the make-up and expression pattern of cellular kinases and downstream effectors in different cells, could differentially regulate signalling outcomes in different cells and tissues by promoting different phosphorylation ‘bar-codes’ on the intracellular face of GPCRs. This might result in the recruitment of distinct adaptor complexes being favoured by unique phosphorylation patterns. To fully explore this hypothesis for FFA1 and/or FFA4 will require a number of steps. Firstly, the identification and characterisation of chemically distinct activating ligands that display ‘bias’ in induced phosphorylation. For FFA4, as described earlier, to date, many of the agonist ligands are based on the phenylpropionic acid core of TUG-891 and hence there is a lack of chemical diversity. Thus, although sites of phosphorylation, induced by such ligands, are now well described (see above), it is most likely that this will be replicated by other synthetic agonist ligands. It may be of considerable help, however, to explore this for fatty acid ligands of different chain length and extent of unsaturation [105]. By contrast, with both described and well characterized orthosteric and allosteric ligands that bind to at least two distinct non-orthosteric sites, it may be that ligands that display bias in FFA1 phosphorylation bar-coding could already exist, even if they have not yet been assessed in this manner. To progress further with this concept, it will, however, require either more widespread and routine application of mass spectrometry to analyse rapidly potential differences in the sites of ligand-induced phosphorylation in FFA1 and FFA4 in a co-ordinated workflow and/or the production and use of a more robust and complete set of ligand-regulated phospho-site specific antisera that cover the full panoply of such sites in FFA4 and in FFA1. These are not yet available for either receptor. Recent times, however, have seen examples of ligand bar-coding for other GPCRs such as the δ-opioid receptor (DOP) where Mann et al. (2020) used phosphosite-specific antibodies at Thr ^361^ and Ser^363^ residues and identified a distinct and hierarchical agonist-induced phosphorylation pattern in the C-terminal tail of DOP [116]. This enabled a definition of a phosphorylation pattern that was aligned with receptor internalisation. Such analysis may support distinct functional effects of DOP ligands within the range of the different functions and effects of this receptor. Further application of such patterns of phosphorylation may allow the identification and characterisation of selective ligands, potentially, those best suited for specific therapeutic applications, and this may then be extended to other GPCRs.

A further potential complication for analysis of FFA4 is that there is an additional FFA4 splice variant in humans [6]. The longer isoform contains an insert of 16 amino acids within the third intracellular loop and this form has been reported to show an intrinsic β-arrestin-bias. Senatorov et al. (2020) recently explored the ability of DHA to promote the phosphorylation of this variant and showed a difference in basal phosphorylation compared to the wild type. Once again however, at least in the background of HEK293 cells, knock-down studies highlighted the important role of GRK6 [137].

**Figure 4:**
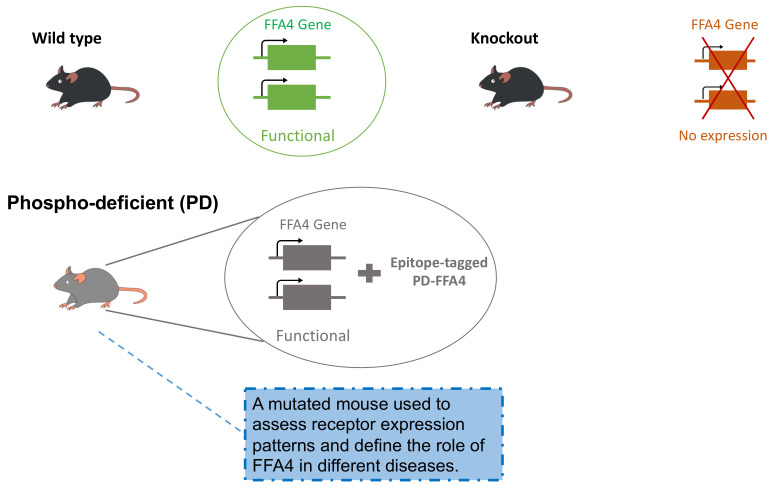
**Production and use of a transgenic knock-in FFA4 phosphorylation deficient mouse line.** After treatment of with an agonist ligand, FFA4 receptors undergo rapid phosphorylation on two sets of Ser/Thr sites located within the intracellular C-tail of the receptor. To explore the role of regulated phosphorylation of FFA4, the wild type receptor was replaced with a variant in which all the hydroxy-amino acids in the C-terminal tail were replaced by alanines (**phospho-deficient, PD**). In addition, to facilitate detection of the expressed receptor protein, the HA-epitope tag sequence was placed in-frame at the C-terminus, as previously reported for the free fatty acid receptor 2 [138,139,140]. Together with the wild type and FFA4 knock-out littermates, a comprehensive understanding of the role of the phosphorylation of FFA4 may be established by studying this line.

Of greater and longer-term importance, all of the studies on the specific nature of GRK subtype regulation of FFA1 and FFA4 have, to date, been performed after heterologous expression of the receptors into simple cell systems. Knock-down/knock-out of GRK isoforms, arrestins and even G proteins, as well as the use of various target and pathway inhibitors, have then allowed analysis of the contribution of specific GRKs and arrestins to the phosphorylation, regulation and signal transduction of a number of GPCRs including FFA1 and FFA4. Ultimately such studies can only hint at the specific roles for these proteins in the regulation of their function in vivo. A start has already been made to explore this through the generation of a transgenic mouse-line, in which FFA4 was replaced by a phospho-deficient (PD) form (Figure 4). Analysis of this line, as well as of a range of other similar transgenic lines that could be produced, will start to provide the levels of insight required to gain a fuller understanding and, hopefully, successful therapeutic programmes targeting these receptors.
